# The role of endothelial nitric oxide in the anti-restenotic effects of liraglutide in a mouse model of restenosis

**DOI:** 10.1186/s12933-017-0603-x

**Published:** 2017-10-02

**Authors:** Hideki Kushima, Yusaku Mori, Masakazu Koshibu, Munenori Hiromura, Kyoko Kohashi, Michishige Terasaki, Tomoyasu Fukui, Tsutomu Hirano

**Affiliations:** Department of Internal Medicine, Division of Diabetes, Metabolism, and Endocrinology, Showa University School of Medicine, 1-5-8 Hatanodai, Shinagawa, Tokyo, 142-8555 Japan

**Keywords:** Endothelium, GLP-1 receptor agonist, Mouse, Nitric oxide, Restenosis

## Abstract

**Background:**

Previous animal studies have shown that glucagon-like peptide-1 receptor agonists (GLP-1RAs) suppress arterial restenosis, a major complication of angioplasty, presumably through their direct action on vascular smooth muscle cells. However, the contribution of vascular endothelial cells (VECs) to this process remains unknown. In addition, the potential interference caused by severe hyperglycemia and optimal treatment regimen remain to be determined.

**Methods:**

Nine-week-old male C57BL6 (wild-type) and diabetic db/db mice were randomly divided into vehicle or liraglutide treatment groups (Day 1), and subject to femoral artery wire injuries (Day 3). The injured arteries were collected on Day 29 for morphometric analysis. Human umbilical vein endothelial cells (HUVECs) were used for in vitro experiments. One-way ANOVA, followed by Tukey’s test, was used for comparisons.

**Results:**

In wild-type mice, liraglutide treatment (5.7, 17, or 107 nmol/kg/day) dose-dependently reduced the neointimal area (20, 50, and 65%) without inducing systemic effects, and caused an associated decrease in the percentage of vascular proliferating cells. However, these effects were completely abolished by the nitric oxide synthase (NOS) inhibitor *N*-omega-nitro-l-arginine methyl ester. Next, we investigated the optimal treatment regimen. Early treatment (Days 1–14) was as effective in reducing the neointimal area and vascular cell proliferation as full treatment (Days 1–29), whereas delayed treatment (Days 15–29) was ineffective. In HUVECs, liraglutide treatment dose-dependently stimulated NO production, which was dependent on GLP-1R, cAMP, cAMP-dependent protein kinase, AMP-activated protein kinase (AMPK), and NOS. Subsequently, we investigated the role of liver kinase B (LKB)-1 in this process. Liraglutide increased the phosphorylation of LKB-1, and siRNA-induced LKB-1 knockdown abolished liraglutide-stimulated NO production. In severe hyperglycemic db/db mice, liraglutide treatment also suppressed neointimal hyperplasia, which was accompanied by reductions in vascular cell proliferation and density. Furthermore, liraglutide treatment suppressed hyperglycemia-enhanced vascular inflammation 7 days after arterial injury.

**Conclusions:**

We demonstrate that endothelial cells are targets of liraglutide, and suppress restenosis via endothelial NO. Furthermore, the protective effects are maintained in severe hyperglycemia. Our findings provide an evidence base for a future clinical trial to determine whether treatment with GLP-1RAs represents potentially effective pharmacological therapy following angioplasty in patients with diabetes.

**Electronic supplementary material:**

The online version of this article (doi:10.1186/s12933-017-0603-x) contains supplementary material, which is available to authorized users.

## Background

Glucagon-like peptide receptor-1 agonists (GLP-1RAs) have been widely used for the treatment of type 2 diabetes owing to their robust glucose-lowering effects, which are accompanied by a reduction in body weight [1]. Recent studies have revealed that GLP-1 is involved in the regulation of multiple physiological processes in various extra-pancreatic tissues, including the cardiovascular system [2]. Preclinical studies, conducted by ourselves and others, have identified the anti-atherogenic effects of GLP-1RAs, which were shown to be partly independent of their metabolic activities [3–9]. Accordingly, two large recent clinical trials, liraglutide effect and action in diabetes: evaluation of cardiovascular outcome results (LEADER) and evaluate cardiovascular and other long-term outcomes with semaglutide in subjects with type 2 diabetes (SUSTAIN-6), have revealed that treatment with GLP-1RAs significantly suppresses major adverse cardiovascular events in patients with type 2 diabetes who are at a high risk for cardiovascular disease. The findings suggest that GLP-1RAs exhibit vascular protective properties [10, 11].

Several animal studies have also shown that GLP-1RAs suppress vascular restenosis [12–15], which shares some pathophysiological similarities with atherosclerosis [16]. Vascular restenosis is one of the major complications of percutaneous transluminal angioplasty (PTA), mainly caused by neointimal hyperplasia, an exaggerated healing process of the damaged intima [17, 18]. Restenosis leads to a narrowing of the artery and ultimately results in additional revascularization. Long-term patency following PTA is markedly improved by the use of drug-eluting stents (DES), which strongly suppress neointimal hyperplasia by inhibiting the proliferation of vascular smooth muscle cells (VSMCs). However, their beneficial effects are reduced in patients with diabetes [19, 20], leaving them at a high risk for restenosis. The suppression of neointimal hyperplasia by GLP-1RAs was evaluated in vivo using mouse femoral artery wire injury and rat carotid artery balloon injury models [12–15]. These models, unlike atherosclerosis models, facilitate the evaluation of the direct vascular effects of an agent in the absence of metabolic abnormalities such as hypercholesterolemia [21]. The direct vascular effects of GLP-1RAs, identified by these studies, support their potential pharmacological use in the treatment of restenosis.

The main pathophysiology of neointimal hyperplasia is VSMC proliferation and migration, resulting from a switch from a contractile to a synthetic phenotype at the site of arterial injury [16]. Previous studies have reported the anti-proliferative and anti-migration effects of GLP-1RAs on VSMCs in vitro [12, 13, 22, 23]. These effects were proposed to underlie the GLP-1RA-mediated suppression of neointimal hyperplasia. However, vascular endothelial cells (VECs) also play an essential role in the suppression of neointimal hyperplasia, and VEC-derived nitric oxide (NO) mediates this effect by inhibiting the phenotypic switching of VSMCs [24–26]. Numerous studies have revealed that GLP-1RAs exert their actions on VECs and enhance endothelial function such as NO production in vitro and NO-dependent vasodilation ex vivo [27–30]. It is known that DES trigger endothelial dysfunction at the site of stenting, leading to restenosis caused by thrombosis and atherosclerosis [24, 25]. Thus, VECs may represent a target for GLP-1RAs in the suppression of restenosis. Therefore, treatment with GLP-1RAs represents potentially effective pharmacological therapy, following PTA with DES. However, the roles of VECs in the anti-restenotic effects of GLP-1RAs remain to be elucidated.

Another question relates to the initiation time and duration of the optimal treatment. In previous animal model studies, GLP-1RA treatment at a single dose was always initiated before arterial injury and continued throughout the experiment. It therefore remains to be determined whether shorter pre- or post-treatment suppresses restenosis. In addition, mildly hyperglycemic rats, with fasting plasma glucose levels of around 180 mg/dL, were only employed in one previous study [15]. Therefore, it remains unclear whether the anti-restenotic effects of GLP-1RAs also occur in the presence of severe hyperglycemia, unlike their actions on pancreatic β cells [31].

In the present study, we demonstrated that treatment with liraglutide dose-dependently suppressed neointimal hyperplasia in vivo; this effect was abrogated by the NO synthase (NOS) inhibitor *N*-omega-nitro-l-arginine methyl ester (l-NAME), indicating that endothelial NO mediates the anti-restenotic effects of liraglutide. Furthermore, liraglutide-stimulated NO production and phosphorylation of endothelial NOS (eNOS) were abolished by liver kinase B1 (LKB1) knockdown in vitro, proposing an essential role of LKB1 in these effects of liraglutide. Moreover, short treatment initiated before, but not after, the injury was as effective in suppressing restenosis as full treatment, and liraglutide exerted anti-restenotic effects in db/db mice with severe hyperglycemia. Our findings justify further studies employing larger animals, such as rabbits and pigs, which would provide better clinical models. Our findings also provide an evidence base for a future clinical trial to determine whether treatment with GLP-1RAs represents potentially effective pharmacological therapy, following PTA with DES in patients with diabetes.

## Methods

In the present study, we aimed to clarify the role of VECs in the anti-restenotic effects of the GLP-1RA liraglutide and examine the underlying mechanism by employing restenosis mouse models and cultures of human VECs. Furthermore, we investigated the efficacy of treatment using different initiation times and durations, and the influence of severe hyperglycemia on the anti-restenotic effects of GLP-1RA.

### Chemical agents

The chemical agents were purchased as follows: liraglutide from Novo Nordisk Japan (Chiyoda, Tokyo, Japan); exendin-(9–39) (Ex-9) from AnaSpec (Fremont, CA, USA); l-NAME, SQ22536 (SQ), cAMP-dependent protein kinase (PKA) inhibitor fragment 14–22 myristoylated trifluoroacetate salt (PKI[14–22]), ESI-09, STO-609, and *N*-omega-nitro-l-arginine methyl ester (l-NAME) from Sigma-Aldrich Japan (Shinagawa, Tokyo, Japan); 10-(4′-[[*N*-diethylamino]]butyl)-2-chlorophenoxazine (Akt inhibitor X) was purchased from Santa Cruz (Dallas, Texas, USA) and dorsomorphin dihydrochloride from Wako (Osaka, Osaka, Japan).

### Animal experiments

**Fig. 1:**
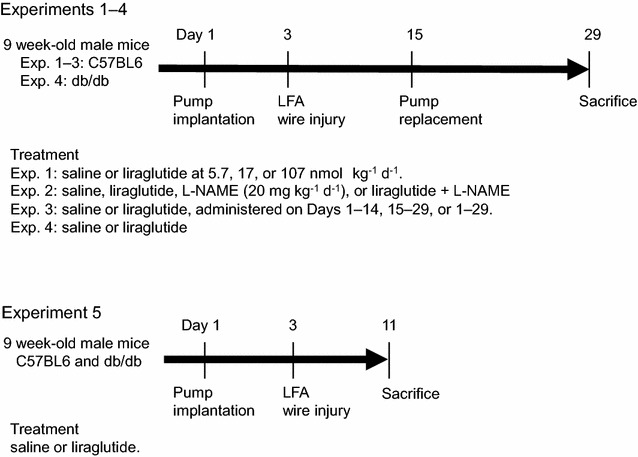
Design of animal experiments (1–5). The liraglutide dose was 17 nmol/kg/day, unless otherwise specified. *N*-omega-nitro-l-arginine methyl ester (l-NAME) was administered via drinking water at the dose of 20 mg/kg/day; LFA, left femoral artery

The study design was approved by the animal care committee of Showa University School of Medicine, and animal experiments were conducted in strict adherence to the Guide for the Care and Use of Laboratory Animals (8th edition, 2011; Office of Laboratory Animal Welfare, National Institutes of Health, MD, USA). All invasive procedures were performed under general anesthesia using isoflurane. Seven-week-old male C57BL6J (wild-type) and db/db mice were purchased from Sankyo Labo Service (Edogawa, Tokyo, Japan) and housed in clean cages (3 mice per cage, maximum) in the Division of Animal Experimentation of Showa University School of Medicine. After a 2-week acclimatization period, mice were randomly divided into vehicle or liraglutide treatment groups. We conducted five animal experiments. The experimental design and treatment groups are shown in Fig. 1. The dose of liraglutide was 17 nmol/kg/day, unless otherwise specified [3, 7, 8]. Agents were delivered using osmotic pumps (Alzet minipump 1002; Cupertino, CA, USA) subcutaneously implanted on Day 1, and the pumps were replaced on Day 15 to avoid liraglutide degradation. l-NAME (20 mg/kg/day), a NOS inhibitor, was administered via drinking water [26]. On Day 3, mice underwent left femoral artery wire injury, as previously reported by Sata and co-workers, with a few modifications [21]. Briefly, the left femoral artery was carefully isolated from the surrounding connective tissues through a small skin incision made in the left inguinal region. A straight spring wire (0.38-mm diameter, No. C-SF-15-15; Cook Japan, Nakano, Tokyo, Japan) was retrogradely inserted into the femoral artery via a small cut on the muscle branch. The wire was inserted to a depth of 7.5 mm and withdrawn after 1 min. The muscle branch was ligated proximal to the cut to prevent bleeding, and the skin incision was sutured. Xylocaine (1%) was added dropwise to the incision area to maintain tissue hydration keep tissues wet and relieve pain. Following the procedure, mice recovered in a separate cage for 6 h under careful observation, after which they were returned to their original cages. The mice did not show any signs of severe complications such as claudication or necrosis of the injured extremity, surgical site infection, or massive weight loss (20% of the baseline). Mice were killed by an overdose of inhaled isoflurane, and the injured arteries (5.5 mm long, from the site of the ligation) were collected on Day 29 for morphometric analyses (experiments 1–4) or on Day 11 for gene expression analyses (experiment 5).

### Assessment of plasma levels

Blood samples were collected after 6–8 h fasting. Plasma glucose levels were measured using a dextrometer (Stat strip XP2, Nipro, Osaka, Osaka, Japan). Hemoglobin A1c (HbA1c) and plasma lipid levels were determined using a latex-enhanced immunoassay and an enzymatic colorimetric assay, respectively (Cobas, Roche Diagnostics Japan, Minato, Tokyo, Japan). Plasma active GLP-1 levels were measured by ELISA (High Sensitivity GLP-1 Active ELISA Kit, EMD Millipore, Billerica, MA, USA).

### Measurement of blood pressure

Blood pressure and pulse rates were measured at the end of the experiments, using the tail-cuff method (Model MK-2000ST; Muromachi Kikai, Chuo, Tokyo, Japan). The average value obtained from 3 to 5 consecutive measurements was used as a single data point.

### Morphometric analysis

Injured arteries were collected for morphometric analysis following perfusion fixation with PBS and 4% paraformaldehyde. Three serial cross-sections taken from the proximal end of the paraffin-embedded femoral arteries at 0.5-mm intervals were stained with Elastica van Gieson (EVG), and the average of three serial cross-sections was used as a single data point. The sections were digitized using an inverted microscope (Model IX71, Olympus, Shinjyuku, Tokyo, Japan) and analyzed using Image J software (National Institutes of Health, Bethesda, MD, USA) by an investigator blinded to the treatment. The areas within the internal elastic lamina and the external laminal perimeter were defined as the neointima and the artery perimeter. Arteries completely occluded by the thrombus were excluded from analysis (Additional file 1: Figure S1a–c). Sections showing the following features were also excluded from analysis (Additional file 1: Figure S1d–f): broken wall structure, a branch of another artery, and missing elastic lamina. The percentage of excluded arteries and sections in each treatment group was approximately 10–20 and 15–25%, respectively. Fisher’s exact test revealed no difference between the groups.

### Immunohistochemistry

Proliferating and endothelial cells were identified by immunostaining using an anti-Ki67 antibody (Thermo Fisher Scientific Japan, Yokohama, Kanagawa, Japan, RM-9106-S1, RRID: AB_149792, raised in rabbit, 1: 250) and an anti-CD31 antibody (Abcam Japan, Chuo, Tokyo, Japan, ab28364, RRID: AB_726362, raised in rabbit, 1: 200), respectively. Nuclei were counterstained with hematoxylin. The averages of three serial cross-sections were used as single data points.

### Reverse transcription-polymerase chain reaction (RT-PCR) analysis

In experiment 5, uninjured and injured femoral arteries were harvested 7 days after injury (Day 11). Total RNA extracted from the arteries was used for synthesizing complementary DNA as previously described [32]. Gene expression was assessed by real-time RT-PCR using the TaqMan gene expression assay and sequence detection system (ABI PRISM 7900; Life Technologies Japan, Minato, Tokyo, Japan). The following pre-designed TaqMan probe sets were used: Glp-1r, Mm00445292_m1; interleukin (*Il)*-*1*, Mm00434228_m1; *Il*-*6*, Mm00446190_m1; tumor necrosis factor (*Tnf)*-*α*, Mm00443258_m1; monocyte chemotactic protein (*Mcp*)-1, Mm00441242_m1; transforming growth factor (*Tgf)*-*β*, Mm01178820_m1. The 18S ribosomal RNA probe (*18sRNA*, Mm03928990_g1) was used as an internal control.

### Cell culture

Human umbilical vein endothelial cells (HUVECs) were obtained from Lonza Japan (Chuo, Tokyo, Japan) and cultured in EBM-2 medium supplemented with FBS and growth factors (Lonza Japan). Collagen I-coated flasks or plates were used for all cell culture experiments. The cells were seeded onto 24-well plates at the following density per well: 2.5 × 10^4^ for NO measurements, 1.0 × 10^4^ for cell proliferation assays, and 4 × 10^4^ for protein extraction and siRNA transfection. Cells were starved in M199 containing 0.3% FBS for 1 h for NO measurements, and in 0.1% FBS containing EBM-2 (without growth factors) for 6 h for protein extraction. For cell culture experiments involving treatment with high levels of glucose, cells were exposed to 25 mmol/L glucose for 48 h before seeding. Cells between passages 4–8 were used for the experiments.

### Measurement of NO production

Plasma NO levels and NO production by HUVECs was determined by measuring the stable metabolites of NO (NO_2_ and NO_3_) in the culture medium (Nitrate/Nitrite Fluorometric Assay Kit; Dojin, Kamimashiki, Kumamoto, Japan) [33]. Protein in plasma samples was removed by ultrafiltration (Pierce concentrator, PES, 10 K MWCO; Thermo Fisher Scientific Japan) before measurement. HUVECs were stimulated with the indicated concentrations of agents (saline or liraglutide 0.1–100 nmol/L) for 2 h, following serum starvation in M199 with 0.3% FBS for 1 h. Inhibitors were added 30 min prior to stimulation at the following concentrations: GLP-1R antagonist Ex-9, 100 nmol/L; cAMP inhibitor SQ, 100 μmol/L; PKA inhibitor PKI (14–22), 1 μmol/L; exchange factor directly activated by cAMP (EPAC) inhibitor ESI-09, 6.4 μmol/L; calcium-calmodulin-dependent protein kinase kinase (CaMKK) inhibitor STO-609, 10 μmol/L; AMP-activated protein kinase (AMPK) inhibitor dorsomorphine, 10 μmol/L; Akt inhibitor X, 5 μmol/L; NOS inhibitor l-NAME, 1 mmol/L [3, 27, 34–39]. The NO_2_ in the plasma samples and medium was converted to NO_3_ by NO_2_ reductase after stimulation, and the medium was incubated with fluorescent 2,3-diaminonaphthalene for 15 min. The fluorescence intensities were measured using an Infinite M200 PRO instrument (TECAN Japan, Kawasaki, Kanagawa, Japan).

### Western blot

Proteins were extracted from HUVECs or mouse aortas using CelLytic M, containing cocktails of protease inhibitors and phosphatase inhibitors (all from Sigma-Aldrich Japan). Western blot was performed as previously described [40]. Briefly, 7 μg of protein per lane was electrophoresed in polyacrylamide gels and transferred to polyvinylidene fluoride membranes. The membranes were blocked with PVDF Blocking Reagent (TOYOBO, Osaka, Osaka, Japan) for 1 h, and then incubated overnight with antibodies at the following dilutions: phosphorylated LKB1 at Ser 428 (p-LKB1; Cell Signaling Technology Japan, Chiyoda, Tokyo, Japan, #3482, RRID: AB_2198321, raised in rabbit, 1: 2500), LKB1 (Cell Signaling Technology Japan, #3050, RRID: AB_823559, raised in rabbit, 1: 5000), p-AMPKα at Thr 178 (p-AMPK; Cell Signaling Technology Japan, #2535, RRID: AB_331250, raised in rabbit, 1: 2500), AMPKα (Cell Signaling Technology Japan, #2532, RRID: AB_330331, raised in rabbit, 1: 5000), p-eNOS at Ser 1177 (p-eNOS; Cell Signaling Technology Japan, #9570, RRID: AB_2298588, raised in rabbit, 1: 2500), eNOS (Cell Signaling Technology Japan, #32,027, RRID: not available, raised in rabbit, 1: 5000), and β-actin (Sigma-Aldrich Japan, A2066, RRID: AB_476693, raised in rabbit, 1: 8000). Goat anti-rabbit IgG conjugated with horseradish peroxidase (GE Healthcare Japan, Minato, Tokyo, Japan, NA934, RRID: AB_772206, raised in donkey) was used at a 1: 40,000 dilution. The Can Get Signal® Immunoreaction Enhancer Solution (TOYOBO) was used for antibody dilutions. The bands on the immunoblot were detected using the Amersham ECL Prime kit (GE Healthcare Japan) and quantified using Image J software.

### siRNA transfection

Predesigned siRNAs for the negative control and LKB1 were purchased from Applied Biosystems (Silencer® Select Negative Control No. 2 siRNA; LKB1 siRNA, S74498; Foster City, CA, USA). The siRNAs were incubated for 30 min with the TransIT-TKO transfection reagent (Mirus Bio, Madison, WI, USA) in serum-free EBM-2, and the mixture was added to the medium at a final siRNA concentration of 25 nmol/L. HUVECs were used for experiments 72 h post-transfection.

### Statistical analysis

Data are expressed as mean ± SEM. We conducted comparisons of more than two groups using a one-way ANOVA, followed by Tukey’s test, and comparisons involving two groups using an unpaired *t* test. Correlations were determined using Pearson’s correlation coefficient test. The Jonckheere-Terpstra trend test was used for determining dose–effect relationships. Statistical calculations were performed using JMP software (version 12; SAS Institute Inc., NC, USA), except for the Jonckheere-Terpstra trend test, which was conducted with R software (Ver 3.2.2; Welthandelsplatz, Vienna, Austria). The significance level was defined at p < 0.05.

## Results

### Liraglutide dose-dependently suppresses neointimal hyperplasia after arterial injury

**Fig. 2:**
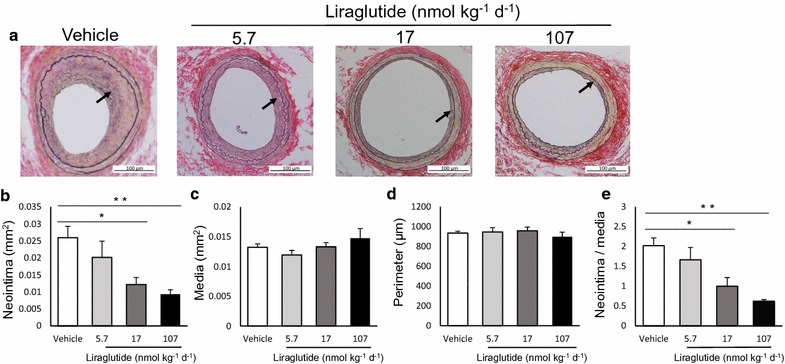
Liraglutide dose-dependently suppresses neointimal hyperplasia. Wild-type mice treated with vehicle or liraglutide at different doses were subject to femoral artery wire injury. The arteries were collected for morphometric analysis 26 days after injury. Cell density was calculated as the number of total cells divided by the area; **a** representative images of cross-sections of femoral arteries; Elastica van Gieson (EVG) staining, 200 ×; **b** neointimal area; **c** medial area; **d** arterial perimeter; **e** intima to media (I/M) ratio. The averages of three serial cross-sections were used as single data points. Arrows indicate the neointima; vehicle and liraglutide at 5.7 and 107 nmol/kg/day, n = 5; liraglutide at 17 nmol/kg/day, n = 6; *p < 0.05; **p < 0.01

First, we investigated the dose–effect relationship of liraglutide against restenosis after arterial injury (animal experiment 1). Wild-type C57BL6 mice were treated with vehicle or increasing doses of liraglutide (5.7, 17, or 107 nmol/kg/day). The physiological and biochemical parameters measured are shown in Table 1. No differences were detected between the groups, except for elevated levels of plasma active GLP-1 in groups treated with liraglutide. When evaluating morphometric changes, liraglutide treatment at 17 and 107 nmol/kg/day significantly suppressed neointimal hyperplasia without inducing medial thinning or arterial dilation. These changes resulted in reductions in the intima to media (I/M) ratio. In contrast, treatment with a 5.7 nmol/kg/day dose of liraglutide did not suppress neointimal hyperplasia (Fig. 2a–e). The Jonckheere-Terpstra trend test revealed a significant trend between the decreases in neointimal area and the increases in liraglutide doses (p < 0.001).

**Table 1:** Physiological and biochemical parameters of mice treated with vehicle or different doses of liraglutide

	Vehicle	Liraglutide (nmol/kg/day)		
		5.7	17	107
Number	5	5	6	5
Food intake (g/day)	4.2 ± 0.2	4.1 ± 0.1	3.8 ± 0.2	4.2 ± 0.1
Water intake (g/day)	4.9 ± 0.2	5.2 ± 0.3	5.7 ± 0.1	4.9 ± 0.2
Initial BW (g)	21.6 ± 0.5	22.6 ± 0.4	22.9 ± 0.2	22.5 ± 0.5
Final BW (g)	22.5 ± 0.6	23.0 ± 0.8	24.8 ± 0.4	25.0 ± 0.4
Pulse rate (beats/min)	704 ± 15	709 ± 10	717 ± 14	707 ± 11
SBP (mmHg)	126 ± 3	123 ± 2	120 ± 5	125 ± 5
DBP (mmHg)	72 ± 7	72 ± 11	66 ± 11	82 ± 11
HbA1c (%)	5.1 ± 0.4	4.8 ± 0.2	4.7 ± 0.2	4.4 ± 0.1
FPG (mg/dL)	111 ± 28	98 ± 9	120 ± 12	112 ± 20
TC (mg/dL)	78 ± 10	67 ± 11	93 ± 5	91 ± 6
TG (mg/dL)	56 ± 3	51 ± 3	48 ± 3	61 ± 3
Active GLP-1 (pmol/L)	1.4 ± 0.2	2.2 ± 0.3	3.6 ± 0.8	8.4 ± 3.9*^†^

Food and water intake were measured throughout the experimental period. The other parameters were evaluated at the end of the experiment, except for initial body weight (BW). Data are expressed as mean ± SEM

*SBP* systolic blood pressure, *DBP* diastolic blood pressure, *FPG* fasting plasma glucose, *TC* total cholesterol, *TG* triglycerides, *GLP-1* glucagon like peptide-1

* p < 0.05 vs. vehicle; ^†^ p < 0.05 vs. liraglutide 5.7 nmol/kg/day

### The anti-restenotic effects of liraglutide are mediated by NO

**Fig. 3:**
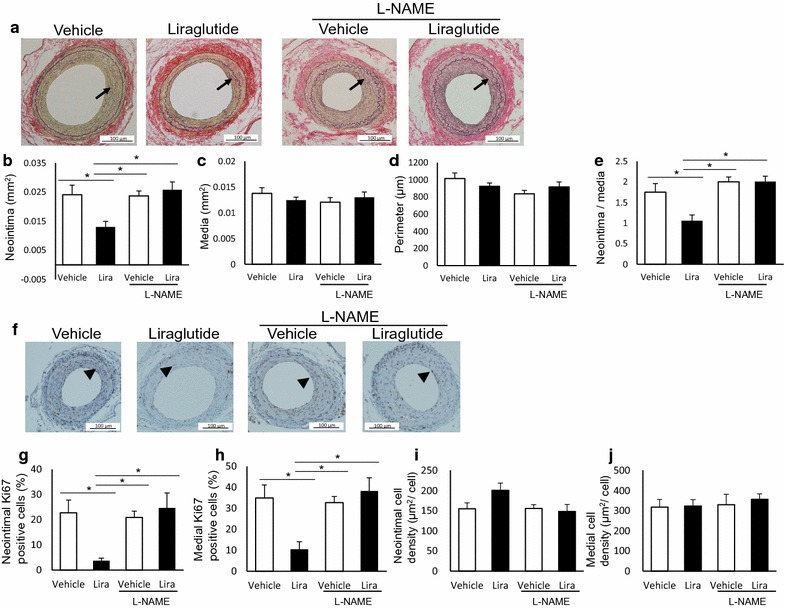
The anti-restenotic effects of liraglutide are mediated by nitric oxide (NO). Wild-type mice were treated with vehicle or liraglutide, with or without the NO synthase (NOS) inhibitor l-NAME. Femoral arteries were collected 26 days after arterial injury. Morphometric changes were evaluated by EVG staining. Cell proliferation and density were assessed by Ki-67 immunostaining and counterstaining. Proliferating cells were defined as cells positive for Ki-67; **a** representative images of cross-sections of femoral arteries (EVG, 200 ×); **b** neointimal area; **c** medial area; d, arterial perimeter; **e** intima to media (I/M) ratio; **f** representative images of injured arteries showing Ki-67 immunostaining (200 ×); **g**, **h** percentage of Ki-67-positive cells to total cells in the neointima and media; **i**, **j**, cell density in the neointima and media. The averages of three serial cross-sections were used as single data points. Arrows indicate the neointima, and arrowheads indicate Ki-67-positive cells; liraglutide, 17 nmol/kg/day; l-NAME, 20 mg/kg/day; n = 5 per group; *p < 0.05

Next, we focused on endothelial NO as a potential mediator of the anti-restenotic effects of liraglutide (animal experiment 2). Vehicle or liraglutide (17 nmol/kg/day) were administered to mice in the presence or absence of the l-NAME NOS inhibitor. In a subset of animals, we observed NOS inactivation by l-NAME treatment in vivo. Plasma NO levels were significantly lower in mice treated with l-NAME than in those treated with vehicle (Additional file 1: Figure S2a). Consistently, l-NAME treatment significantly suppressed phosphorylation of eNOS in the aorta compared to vehicle treatment (Additional file 1: Figure S2b, c). Table 2 shows the physiological and biological parameters of each treatment group. Mice treated with l-NAME exhibited higher systolic blood pressure levels than those not administered the inhibitor, as previously reported [41]. Co-treatment with l-NAME completely abolished the suppression of neointimal hyperplasia by liraglutide, while the medial area and the arterial perimeter were not affected (Fig. 3a–e). Furthermore, liraglutide treatment decreased the percentages of intimal and medial proliferating cells, as assessed by cells that stained positive for the Ki-67 marker; however, these effects were not observed in mice co-treated with l-NAME (Fig. 3f–h). The number of proliferating cells in the neointima and media was correlated with neointimal hyperplasia and medial thinning, respectively (Table 3). In contrast, the density of neointimal or medial cells, calculated as the number of total cells divided by the area, was not affected by treatment with liraglutide or l-NAME (Fig. 3i, j).

**Table 2:** Physiological and biochemical parameters of vehicle- or liraglutide-treated mice with or without *N*-omega-nitro-l-arginine methyl ester

	Vehicle	Liraglutide (17 nmol/kg/day)	+l-NAME (20 mg/kg/day)	
			Vehicle	Liraglutide (17 nmol/kg/day)
Number	5	5	5	5
Food intake (g/day)	4.1 ± 0.1	3.6 ± 0.3	3.8 ± 0.1	4.2 ± 0.1
Water intake (g/day)	4.7 ± 0.3	5.3 ± 0.5	4.5 ± 0.1	5.0 ± 0.1
Initial BW (g)	22.4 ± 0.7	23.3 ± 0.2	21.7 ± 0.3	22.0 ± 0.6
Final BW (g)	22.9 ± 0.6	25.3 ± 1.0	22.5 ± 0.1	23.8 ± 0.9
Pulse rate (beats/min)	702 ± 15	712 ± 16	645 ± 30	660 ± 24
SBP (mmHg)	126 ± 3	120 ± 6	143 ± 3*†	130 ± 3
DBP (mmHg)	72 ± 10	71 ± 13	97 ± 10	80 ± 10
HbA1c (%)	4.6 ± 0.1	4.5 ± 0.1	4.7 ± 0	4.7 ± 0.1
FPG (mg/dL)	121 ± 10	117 ± 8	120 ± 12	120 ± 14
TC (mg/dL)	89 ± 2	83 ± 6	90 ± 3	93 ± 2
TG (mg/dL)	49 ± 1	50 ± 4	54 ± 1	57 ± 2

Food and water intake were measured throughout the experimental period. The other parameters were evaluated at the end of the experiment, except for initial BW

*l* *-NAME N*-omega-nitro-l-arginine methyl ester

* p < 0.05 vs. vehicle; ^†^ p < 0.05 vs. liraglutide

**Table 3:** Correlation of the neointimal and medial areas with the number of proliferating and total cells

Neointimal area	r	p	Medial area	r	p
Combined			Combined		
Intima			Intima		
Ki67+ cells	0.48	< 0.0001	Ki67+ cells	0.04	0.73
Total cells	0.85	< 0.0001	Total cells	− 0.44	< 0.01
Media			Media		
Ki67+ cells	− 0.20	0.12	Ki67+ cells	− 0.30	0.02
Total cells	− 0.21	0.10	Total cells	0.45	< 0.01
Vehicle alone			Vehicle alone		
Intima			Intima		
Ki67+ cells	0.68	< 0.01	Ki67+ cells	0.09	0.70
Total cells	0.92	< 0.0001	Total cells	− 0.31	0.19
Media			Media		
Ki67+ cells	− 0.07	0.79	Ki67+ cells	0.00	0.99
Total cells	0.13	0.60	Total cells	0.46	0.04
Liraglutide alone			Liraglutide alone		
Intima			Intima		
Ki67+ cells	0.61	< 0.01	Ki67+ cells	0.37	0.06
Total cells	0.88	< 0.0001	Total cells	− 0.35	0.08
Media			Media		
Ki67+ cells	− 0.39	0.05	Ki67+ cells	0.00	0.98
Total cells	− 0.39	0.05	Total cells	0.69	< 0.0001

r indicates Pearson’s correlation coefficient; Combined, 63 sections taken from 31 mice; vehicle alone, 20 sections taken from 10 mice; liraglutide alone, 26 sections taken from 11 mice

### Early, but not delayed, treatment with liraglutide is as effective in the suppression of restenosis as full treatment

**Fig. 4:**
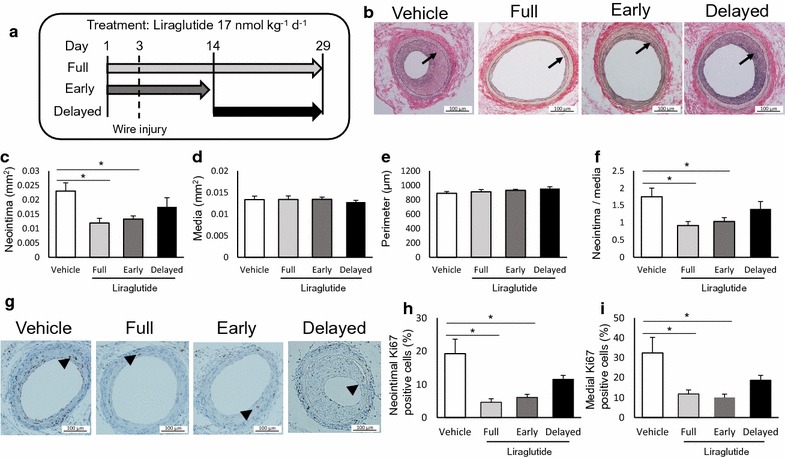
Early short, but not delayed, treatment with liraglutide is effective in suppressing restenosis. Liraglutide (17 nmol/kg/day) was administered to mice at different initiation times and durations: Days 1–29 (full), Days 1–14 (Early), and Days 15–29 (Delayed). Changes in morphometry and cell proliferation were evaluated on Day 29; **a** scheme of the experimental design; **b** representative images of cross-sections of femoral arteries (EVG, 200 ×); **c** neointimal area; **d** medial area; **e** arterial perimeter; **f** intima to media (I/M) ratio; **g** representative images of injured arteries showing Ki-67 immunostaining (200 ×); **h**, **i** percentage of Ki-67-positive cells to total cells in the neointima and media, respectively. The averages of three serial cross-sections were used as single data points. Arrows indicate the neointima, and arrowheads indicate Ki-67-positive cells; **d**–**f**: n = 6 for vehicle and full treatment; n = 9 for early short treatment; n = 8 for delayed treatment; **i**, **j**: n = 5 per group; *p < 0.05

To determine the optimal treatment regimen, we compared the anti-restenotic effects of early and delayed treatment (Days 1–14 and Days 15–29 days, respectively) with those achieved by full treatment (Day 1–29) (animal experiment 3). Figure 4a shows the experimental design. The formation of the neointima and endothelial regeneration, at different time points after arterial injury, are presented in Additional file 1: Figure S3. On Day 14, at the beginning of the delayed treatment, the neointima was not clearly noticeable; however, endothelial regeneration, as assessed by the CD31-positive area, was almost complete. Physiological and biochemical parameters were comparable between the groups (Table 4). Although treatment was terminated at Day 14, the early treatment group exhibited suppression of neointimal hyperplasia on Day 29, which was similar to that observed in the full treatment group (Fig. 4b–f). In contrast, this suppression was reduced in the delayed treatment group. Furthermore, early treatment was effective in reducing the proliferation of vascular cells, as assessed on Day 29 (Fig. 4g–i). Similar to neointimal hyperplasia, delayed treatment failed to suppress the proliferation of vascular cells.

**Table 4:** Physiological and biochemical parameters of mice treated with vehicle or liraglutide

	Vehicle	Liraglutide (17 nmol/kg/day)		
		Full	Early	Delayed
Number	6	6	9	8
Initial BW (g)	21.2 ± 0.4	21.9 ± 0.1	22.3 ± 0.3	21.9 ± 0.1
Final BW (g)	22.6 ± 0.5	23.3 ± 0.4	23.3 ± 0.3	23.6 ± 0.3
Heart rate (beats/min)	696 ± 28	722 ± 21	722 ± 8	715 ± 22
SBP (mmHg)	124 ± 2	119 ± 6	110 ± 4	115 ± 2
DBP (mmHg)	68 ± 13	66 ± 14	43 ± 6	62 ± 5
HbA1c (%)	4.6 ± 0.2	4.7 ± 0.2	4.7 ± 0.0	4.7 ± 0.1
FPG (mg/dL)	118 ± 7	114 ± 13	138 ± 8	136 ± 10
TC (mg/dL)	92 ± 8	89 ± 4	91 ± 1	91 ± 3
TG (mg/dL)	51 ± 3	52 ± 3	52 ± 3	60 ± 3

Food and water intake were measured throughout the experimental period. The other parameters were evaluated at the end of the experiment, except for initial BW. The initiation time and the duration of liraglutide treatment were as follows: full, Days 1–29; Early, Days 1–14; Delayed, Days 15–29; the experiment design is illustrated in Fig. 3a

### Liraglutide stimulates endothelial NO production via the GLP-1R/cAMP/PKA/AMPK/eNOS pathway in vitro

**Fig. 5:**
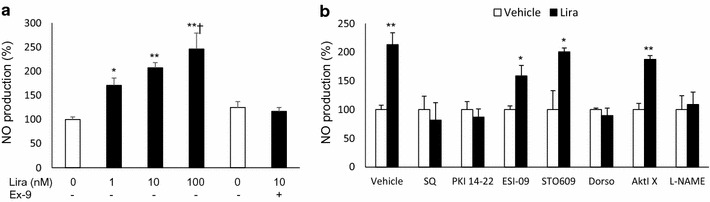
Liraglutide stimulated NO production via the cAMP/PKA/AMPK/eNOS pathway in HUVECs. NO production by HUVECs was determined by measuring levels of the stable metabolites of NO (NO_2_ and NO_3_) in the culture medium. HUVECs were stimulated with the indicated concentrations of agents (saline or liraglutide 0.1–100 nmol/L) for 2 h, following serum starvation in M199, 0.3% FBS, for 1 h. Inhibitors were added 30 min prior to stimulation at the following concentrations: GLP-1R antagonist exendin-(9–39) (Ex-9), 100 nmol/L; cAMP inhibitor SQ22536 (SQ), 100 μmol/L; cAMP-dependent protein kinase (PKA) inhibitor fragment 14–22 myristoylated trifluoroacetate salt (PKI[14–22]), 1 μmol/L; exchange factor directly activated by cAMP inhibitor (ESI-09), 6.4 μmol/L; calcium-calmodulin-dependent protein kinase kinase inhibitor (STO-609), 10 μmol/L; AMP-activated protein kinase (AMPK) inhibitor (dorsomorphine), 10 μmol/L; Akt inhibitor 10-(4′-[[*N*-diethylamino]]butyl)-2-chlorophenoxazine (AktI X), 5 μmol/L; NOS inhibitor (l-NAME), 1 mmol/L; **a** effects of liraglutide and the Ex-9 GLP-1R antagonist on endothelial NO production, 2 h after stimulation; **b** effects of inhibitors of downstream molecules of GLP-1R on liraglutide-stimulated NO production; n = 4–6; *p < 0.05, ** < 0.01 vs. vehicle; ^†^p < 0.05 vs. liraglutide 1 nmol/L

To investigate the mechanism underlying the observed effects, we examined the molecules involved in liraglutide-stimulated NO production in vitro. Liraglutide treatment dose-dependently stimulated NO production in HUVECs, which was blocked by the Ex-9 GLP-1R antagonist (Fig. 5a). This effect was also completely abolished by the cAMP, PKA, AMPK, or NOS inhibitors, but not by the EPAC, CaMKK, or Akt inhibitors (Fig. 5b). Taken together, our data suggest that endothelial NO production induced by liraglutide is dependent on the GLP-1R/cAMP/PKA/AMPK/eNOS pathway in vitro.

### LKB1 is essential for liraglutide-stimulated NO production

**Fig. 6:**
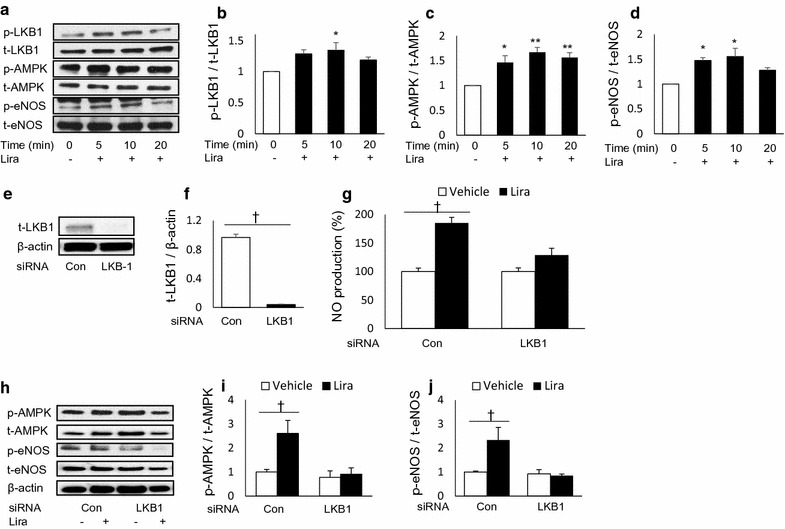
Liver kinase B1 (LKB1) is essential for liraglutide-stimulated NO production in HUVECs. HUVECs were serum-starved with EBM-2 (0.1% FBS and growth factor-free) for 6 h, and proteins were extracted after stimulation with liraglutide (10 nmol/L) for the indicated time; **a** representative western blot images of phosphorylated (p-) and total (t-) LKB1, AMPK, and endothelial NOS (eNOS) at 0, 5, 10, and 20 min; **b**–**d** ratio of p- to t-LKB1, AMPK, and eNOS 20 min after liraglutide stimulation, n = 4–6; *p < 0.05, **p < 0.01 vs. 0 min. Predesigned siRNAs for negative control or LKB1 were transfected to HUVECs at 25 nmol/L. HUVECs were used for experiments 72 h post-transfection; **e**, **f** representative western blot images of LKB1 and β-actin 72 h after siRNA transfection, and ratio of LKB1 to β-actin, n = 4; ^†^p < 0.05; **g** effects of LKB1 knockdown on liraglutide-stimulated NO production; **h**–**j** representative western blot images of siRNA-transfected HUVECs 20 min after liraglutide stimulation, and ratio of p- to t-AMPK and eNOS, n = 5–6; ^†^p < 0.05

Next, we investigated the role of LKB-1 as a molecule that potentially links PKA to AMPK. Liraglutide treatment significantly increased the phosphorylation of LKB1 in HUVECs, consistent with the phosphorylation of AMPK and eNOS (Fig. 6a–d). Cells transfected with siRNA against LKB1 exhibited a ~ 95% decrease in LKB1 protein levels compared with cells transfected with scrambled siRNA (Fig. 6e, f). LKB1 knockdown abrogated the effects of liraglutide on NO production (Fig. 6g). Moreover, LKB1 knockdown inhibited liraglutide-induced phosphorylation of AMPK and eNOS (Fig. 6h–j).

### The anti-restenotic effects of liraglutide are preserved in severely hyperglycemic db/db mice

**Fig. 7:**
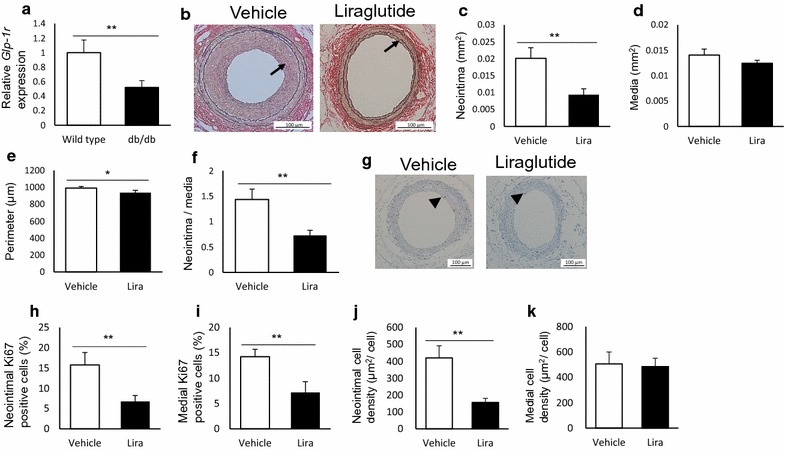
The anti-restenotic effects of liraglutide are preserved in severely hyperglycemic db/db mice. db/db mice with severe hyperglycemia (random plasma glucose levels > 300 mg/dL) were treated with vehicle or liraglutide (17 nmol/kg/day), and subject to femoral artery wire injury. Changes in morphometry and cell proliferation were evaluated 26 days after injury; **a** vascular expression of glucagon like peptide-1 receptor (*Glp*-*1r)* in wild-type and db/db mice at the time of treatment initiation. Gene expression was assessed by real-time reverse transcription-polymerase chain reaction (RT-PCR) at the time of treatment initiation; **b** representative images of cross-sections of femoral arteries (EVG, 200 ×); **c** neointimal area; **d** medial area; **e** arterial perimeter; **f** intima to media (I/M) ratio; **g** representative images of injured arteries with Ki-67 immunostaining (200 ×); **h**, **i** percentage of Ki-67-positive cells to total cells in the neointimal and medial regions; **j**, **k** cell density in the neointima and media. The averages of three serial cross-sections were used as single data points. Arrows indicate the neointima, and arrowheads indicate Ki-67-positive cells, n = 6 per group; *p < 0.05; **p < 0.01

We examined whether the anti-restenotic effects of liraglutide were preserved under severe hyperglycemia in db/db mice, a model of obesity-induced diabetes (animal experiment 4). At the time of treatment initiation (at the age of 9 weeks), all db/db mice exhibited severe hyperglycemia (random plasma glucose levels over 300 mg/dL), and a significantly decreased *Glp*-*1r* expression in the aorta, compared to that in nondiabetic wild-type mice (Fig. 7a). First, we determined the dose of liraglutide to be administered. The body weights and the fasting plasma glucose levels of db/db mice were significantly reduced following liraglutide treatment with 107 nmol/kg/day compared with those of mice administered vehicle treatment, while treatment with 17 nmol/kg/day liraglutide did not affect body weight, and caused a slight decrease in fasting plasma glucose levels (Additional file 1: Figure S4a, b). To avoid the potential influence of systemic effects, we chose a 17 nmol/kg/day dose of liraglutide for this experiment. The physiological and biochemical parameters are presented in Table 5. Fasting plasma glucose and HbA1c levels tended to be lower in liraglutide-treated mice (p = 0.20 and 0.66, respectively). Other parameters were similar between the groups, except for the pulse rate and plasma active GLP-1 levels. Treatment with liraglutide significantly suppressed neointimal hyperplasia compared with that achieved by treatment with the vehicle, without inducing medial thinning or arterial dilation (Fig. 7b–f). In addition, liraglutide treatment reduced the percentage of neointimal and medial proliferating cells (Fig. 7g–i). Further, the reduction in the number of neointimal proliferating cells was correlated with the neointimal area (Table 6). Liraglutide treatment also decreased intimal cell density, which was not changed in normoglycemic mice (Fig. 7j, k).

**Table 5:** Physiological and biochemical parameters of diabetic db/db mice treated with vehicle or liraglutide

	Vehicle	Liraglutide (17 nmol/kg/day)
Number	6	6
Food intake (g/day)	7.6 ± 0.2	6.7 ± 0.3
Water intake (g/dy)	10.2 ± 0.2	9.6 ± 0.2
Initial BW (g)	40.6 ± 0.4	39.5 ± 0.4
Final BW (g)	45.5 ± 0.9	43.9 ± 0.7
BW change (g)	4.9 ± 0.7	4.4 ± 0.7
Heart rate (beats/min)	584 ± 7	624 ± 7*
SBP (mmHg)	135 ± 8	141 ± 3
DBP (mmHg)	76 ± 7	69 ± 6
HbA1c (%)	9.5 ± 0.3	9.1 ± 0.8
FPG (mg/dL)	504 ± 41	390 ± 69
TC (mg/dL)	121 ± 7	126 ± 3
TG (mg/dL)	117 ± 12	105 ± 6
Active GLP-1 (pmol/L)	4.2 ± 1.0	6.8 ± 0.5*

Food and water intake were measured throughout the experimental period. The other parameters were evaluated at the end of the experiment, except for initial BW

* p < 0.05 vs. vehicle

**Table 6:** Correlations between neointimal and medial areas with proliferating and total cell counts in db/db mice

Neointimal area	r	p	Medial area	r	p
Combined			Combined		
Intima			Intima		
Ki67+ cells	0.80	< 0.0001	Ki67+ cells	0.20	0.32
Total cells	0.83	< 0.0001	Total cells	0.37	0.05
Media			Media		
Ki67+ cells	0.27	0.16	Ki67+ cells	0.38	0.05
Total cells	0.00	0.99	Total cells	0.45	0.01

r indicates Pearson’s correlation coefficient. Twenty-eight sections, taken from 10 mice, were used for analysis

### Liraglutide stimulates endothelial NO production in a hyperglycemic milieu in vitro

**Fig. 8:**
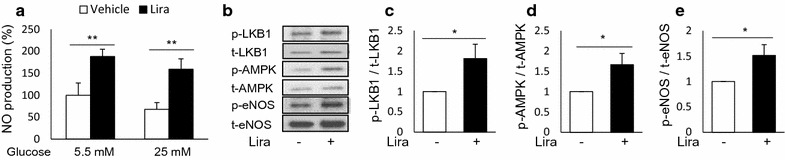
Effects of liraglutide on NO production under hyperglycemic conditions. HUVECs were cultured in normal (5.5 mmol/L) or high-glucose (25 mmol/L) medium 48 h prior to the experiments; **a** NO production 2 h after vehicle or liraglutide (10 nmol/L) stimulation under normal or high glucose conditions, n = 4–6; **b** representative western blot images of HUVECs cultured in high-glucose, stimulated with vehicle or liraglutide (10 nmol/L) for 20 min, and the ratio of p- to t-LKB1 (**c**), AMPK (**d**), and eNOS (**e**), n = 6–8; *p < 0.05; **p < 0.01

We examined the effects of hyperglycemia on liraglutide-stimulated endothelial NO production in vitro. HUVECs were exposed to a high glucose concentration (25 mmol/L) 96 h before the experiments. Liraglutide treatment significantly increased NO production in HUVECs cultured in high concentrations of glucose, as well as in those cultured under normal glucose conditions (Fig. 8a). Similarly, liraglutide treatment significantly increased the phosphorylation of LKB1, AMPK, and eNOS in HUVECs cultured in high-glucose media (Fig. 8b–e).

### Liraglutide suppresses the hyperglycemia-enhanced vascular expression of pro-inflammatory cytokines and *Tgf*-*β* after arterial injury

**Fig. 9:**
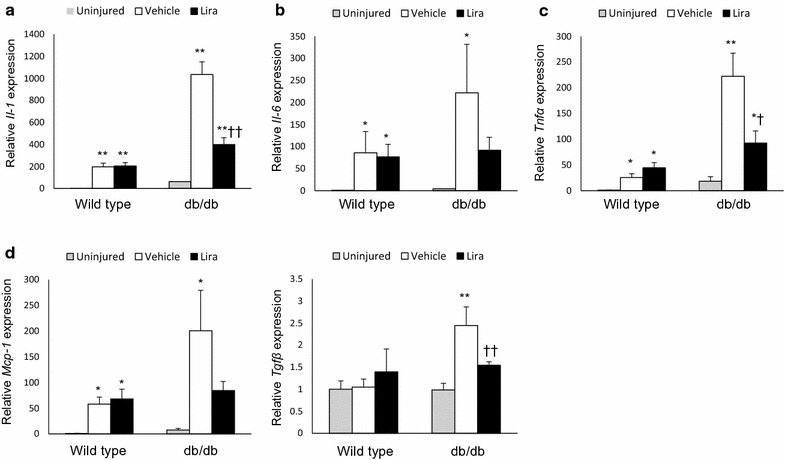
Liraglutide suppressed the hyperglycemia-enhanced vascular expression of pro-inflammatory cytokines and growth factors after arterial injury. Uninjured and injured femoral arteries of wild-type and db/db mice were collected 7 days after wire injury. Gene expression was assessed by real-time RT-PCR; **a**–**e** vascular expression of *Il*-*1, Il*-*6, Mcp*-*1, Tnf*-*α,* and *Tgf*-*β*. Data represent the relative expression levels of target genes normalized to that of 18S rRNA, n = 6–8 per group; *p < 0.05, **p < 0.01 vs. uninjured artery; ^†^p < 0.05, ^††^p < 0.01 vs. vehicle; *Il*, interleukin; *Mcp*-*1*, monocyte chemotactic protein; *Tnf*, tumor necrosis factor; *Tgf*, transforming growth factor

Finally, we investigated the anti-inflammatory effects of liraglutide in vivo (animal experiment 5). Femoral arteries were collected from a different set of animals 7 days after injury (Day 11). In normoglycemic wild-type mice, liraglutide did not affect the injury-induced vascular expression of pro-inflammatory cytokines, chemokines, or growth factors (Fig. 9a–e). In hyperglycemic db/db mice, inflammatory responses to the injury were substantially enhanced compared with those in normoglycemic mice. Further, liraglutide treatment significantly suppressed the hyperglycemia-enhanced vascular expression of *Il*-*1*, *Tnf*-*α*, and *Tgf*-*β*, and tended to reduce *Il*-*6* and *Mcp*-*1* levels (Fig. 9a–e).

## Discussion

In the present study, we demonstrate for the first time that the anti-restenotic effects of liraglutide are abrogated by the l-NAME NOS inhibitor in vivo, and that LKB1 is essential for liraglutide-stimulated endothelial NO production in vitro. We additionally found that treatment initiated before, but not after, injury was as effective in suppressing restenosis as full treatment, and that liraglutide exerts anti-restenotic effects in db/db mice with severe hyperglycemia.

Moreover, we demonstrated that liraglutide suppresses restenosis after arterial injury, consistently with previous animal studies [12–15]. Importantly, we identified a novel, clear dose–effect relationship between the dose of liraglutide and neointimal hyperplasia. Previous studies have shown dose-dependent suppression of the formation of atherosclerotic lesions following GLP-1RA treatment in mouse models of atherosclerosis [6]. Our findings suggest that a higher dose of GLP-1RA should be considered for the suppression of not only atherosclerosis but also restenosis. Notably, all vascular protective effects of liraglutide, including the suppression of vascular cell proliferation, were completely abolished by treatment with the l-NAME NOS inhibitor. Interestingly, the protective effects of endothelial NO against vascular remodeling are supported by several previous studies [24–26, 42]. Treatment with NO donors and transfection of the eNOS gene to the injured site have been shown to suppress restenosis in animal models [43, 44]. Thus, endothelial NO may play a crucial role in the liraglutide-induced suppression of neointimal hyperplasia and associated vascular cell proliferation.

We additionally sought to determine the optimal treatment initiation time and the duration of liraglutide administration. Both early and delayed treatments were initiated before neointimal formation, and the treatment period for each was the same (14 days). However, the two treatments led to different vascular outcomes. Early treatment was as effective in preventing neointimal hyperplasia as full treatment, while the delayed treatment failed to accomplish this. Similar effects for liraglutide against atherosclerosis have been reported in mice: liraglutide suppressed the formation of atheromatous plaque in younger apolipoprotein-null mice, but failed to suppress the progression of it in older mice [8]. Therefore, the effectiveness of the anti-restenotic and atherogenic outcomes of liraglutide treatment may be limited to the early phase of restenosis and atherosclerosis. Moreover, the findings indicate that liraglutide treatment should be initiated as early as possible to obtain maximal vasoprotective effect.

Our findings also provide insights into the source of endothelial NO responsible for the anti-restenotic effects of liraglutide. Injured arteries were completely denuded of endothelial cells as a result of wire injury in our model [21]. Although the denuded lumen is gradually covered by regenerating endothelial cells, this regeneration process was not completed during the early, short treatment. Thus, it is reasonable to assume that the endothelial NO is derived from intact endothelial cells from uninjured arteries.

It has been argued whether VECs express functional GLP-1Rs. Several studies support the direct action of GLP-1RAs on endothelial cells. GLP-1RAs have been shown to increase the endothelium-dependent vasodilation ex vivo, and endothelial NO production in vivo [27–30]. Although the GLP-1R/cAMP/PKA/AMPK/eNOS pathway has been proposed to underlie these effects, the molecule that links PKA and AMPK has not been identified. A previous study has demonstrated the PKA-dependent phosphorylation of LKB1 by GLP-1RA in VECs [30]; however, it remains unclear whether this effect is associated with the activation of AMPK. To address this, we first confirmed the previous findings: the liraglutide-stimulated endothelial NO production was blocked by inhibiting GLP-1R, cAMP, PKA, AMPK, and eNOS in HUVECs. Next, we demonstrated that LKB-1 knockdown inhibited liraglutide-stimulated NO production and AMPK phosphorylation. Conversely, liraglutide-stimulated NO production was not affected by the inhibition of CaMKK-β, another molecule located upstream of AMPK [38]. Our findings indicate that LKB1 is essential for the enhancement of NO production by liraglutide, and acts upstream of AMPK.

Another aim of the present study was to determine whether the anti-restenotic effects of liraglutide were preserved under chronic severe hyperglycemia using db/db mice, a model of obesity-induced diabetes. It has been reported that chronic hyperglycemia leads to impaired GLP-1 activity in pancreatic β cells [31, 45]; however, evidence regarding the vascular role of GLP-1 under these conditions is limited, except for several studies in mice with mild hyperglycemia [15, 46]. Unique to this study, and to minimize the influence of systemic effects, liraglutide was administered to db/db mice at a dose that did not induce weight loss. Although the vascular expression of GLP-1R decreased in severely hyperglycemic db/db mice, liraglutide suppressed neointimal hyperplasia, similar to its effect in normoglycemic wild-type mice. Interestingly, the effects of liraglutide on vascular cell proliferation and density after arterial injury differed between normoglycemic and hyperglycemic mice: in hyperglycemic mice, the suppression of vascular cell proliferation following liraglutide treatment was weaker than that in normoglycemic mice (60% vs. 80%); however, liraglutide treatment reduced vascular cell density in hyperglycemic, but not in normoglycemic mice (60% reduction). Cell density is associated with the deposition of the extracellular matrix (ECM), which contributes to the progression of neointimal hyperplasia. Liraglutide treatment also attenuated the injury-induced vascular expression of proinflammatory cytokines involved in vascular remodeling in hyperglycemic mice [47]. In addition, liraglutide treatment reduced the hyperglycemia-enhanced expression of *Tgf*-*β*, which is associated with vascular complications in patients with diabetes [48, 49]; this change is consistent with the reduction in cell density. This suppression of inflammation and vascular ECM deposition may have contributed to the anti-restenotic effects of liraglutide in hyperglycemia.

Liraglutide treatment has been shown to reduce body weight gain in animals and humans. In the present study, liraglutide treatment at the high dose (107 nmol/kg/day), but not the low dose (17 nmol/kg/day), significantly reduced body weight gain in db/db mice; however, this effect of liraglutide was not observed in wild-type C57BL6 mice. It is possible that surgical stress from osmotic pump implantation and femoral artery wire injury could have masked this effect of liraglutide. C57BL6 mice that received no surgical stress showed body weight gains of approximately 2–4 g/month. Meanwhile, the mice that received osmotic pump implantation and femoral artery wire injury showed body weight gains of only approximately 0.5–1.5 g/month. Thus, liraglutide treatment did not induce further body weight reduction under conditions of surgical stress.

One of the limitations of the present study was the use of l-NAME to inhibit endothelial NO production in vivo. As l-NAME is a non-specific NOS inhibitor, it may have masked the potential effects of liraglutide on other NOS isoforms: the inducible and the neuronal NOS [50]. In addition, eNOS in VSMCs may be involved in the anti-restenotic effects of liraglutide. eNOS is expressed exclusively in ECs. However, recent studies have demonstrated that VSMCs express eNOS under hypoxic conditions via promoter demethylation [51], and that liraglutide increases eNOS expression in cultured rat pulmonary artery smooth muscle cells [52]. For a better understanding of the role of eNOS at the molecular level, a study employing cell-specific gene deletion, including an inducible Cre-loxP or tetracycline-controlled transcriptional regulation system, is required [53, 54]. Moreover, it remains unclear whether liraglutide exerts additive anti-restenotic effects in DES. Our findings justify further studies employing larger animals, such as rabbits and pigs, which would provide better clinical models. Another concern is potential adverse effects of high-dose liraglutide on the cardiovascular system. It is reported that treatment with GLP-1RAs including liraglutide increases heart rate in animal and humans [10, 11, 55–57]. In addition, liraglutide treatment has been shown not to improve the systolic function of the left ventricle in patients with type 2 diabetes and stable coronary artery disease, indicating that liraglutide treatment exerts positive chronotropic, but not inotropic effects on the heart [58]. Thus, exceedingly high levels of liraglutide could result in adverse effects on the cardiovascular system by positive chronotropic effects in acute settings. Our findings indicate that a higher dose of liraglutide is more effective at suppressing restenosis; however, an optimum dose of liraglutide balancing anti-restenotic and positive chronotropic effects needs to be determined.

## Conclusions

In conclusion, we demonstrate that endothelial cells are the target of liraglutide, which suppresses restenosis via endothelial NO. This anti-restenotic effect is retained under severe hyperglycemia, and earlier initiation of GLP-1RA treatment may lead to a better vascular outcome. To our knowledge, the present study is the first to report that the anti-restenotic effects of liraglutide are abolished by the l-NAME NOS inhibitor in vivo, and that LKB1 is essential for liraglutide-stimulated endothelial NO production in vitro. Our findings provide an evidence base for a future clinical trial to determine whether treatment with GLP-1RAs represents potentially effective pharmacological therapy, following PTA with DES in patients with diabetes.

## Additional file

**Additional file 1.** Additional figures (Figs. S1–S4).

## Abbreviations

cAMP: cyclic adenosine monophosphate
AMPK: AMP-activated protein kinase
CaMKK: calcium-calmodulin-dependent protein kinase kinase
eNOS: endothelial nitric oxide synthase
DES: drug eluting stent
DPP-4: dipeptidyl peptidase-4
EPAC: exchange factor directly activated by cAMP
EVG: Elastica van Gieson
Ex-9: exendin-(9–39)
GLP-1: glucagon like peptide-1
GLP-1RA: glucagon like peptide-1 receptor agonist
HbA1c: hemoglobin A1c
HG: high glucose
HUVEC: human umbilical vein endothelial cell
Il: interleukin
I/M ratio: intima/media ratio
LKB1: liver kinase B1
l-NAME: *N*-omega-nitro-l-arginine methyl ester
MCP: monocyte chemotactic protein
NG: normal glucose
NO: nitric oxide
PDGF: platelet-derived growth factor
PKA: cyclic AMP-dependent protein kinase
PTA: percutaneous transluminal angioplasty
RT-PCR: reverse transcription-polymerase chain reaction
siRNA: small interfering RNA
TGF: transforming growth factor
TNF: tumor necrosis factor
VEC: vascular endothelial cell
VSMC: vascular smooth muscle cell
WST: water soluble tetrazolium
18S rRNA: 18S ribosomal RNA
