# Supplementary material for: The role of endothelial nitric oxide in the anti-restenotic effects of liraglutide in a mouse model of restenosis
Source: Cardiovasc Diabetol. 2017 Oct 2;16:122. doi: 10.1186/s12933-017-0603-x (PMC5625638; doi:10.1186/s12933-017-0603-x)
Supplement: Supplementary file 1 — Additional file 1. Additional figures (Figs. S1–S4). [file 12933_2017_603_MOESM1_ESM.docx]

Fig. S1. Exclusion criteria of arteries and sections. The arteries were collected after perfusion-fixation at Day 29.

a, injured artery without thrombus. Arteries completely occluded by thrombus were excluded from analysis: b, injured artery with thrombus; c, cross-section of the artery occluded by thrombus (EVG, 200x). Cross-sections showing the following characteristics were excluded from analysis: d, broken wall structure; e, a branch of another artery; f, missing internal elastic lamina.

Fig. S2. N-omega-nitro-L-arginine methyl ester (L-NAME) treatment inhibits nitric oxide synthase (NOS) activity *in vivo*. Plasma samples and aortas were collected from C57BL6 mice treated with vehicle or L-NAME (20 mg kg^−1^ d^−1^ via drinking water) for 7 d.

a, Plasma NO levels, n=10 for vehicle and n=7 for L-NAME; b, representative western blot images of phosphorylated (p-) and total (t-) endothelial NOS (eNOS) and β-actin; c, the ratio of p- to t-eNOS, n = 3; *, p < 0.05.

Fig. S3. Neointimal formation and endothelial regeneration at different time points after arterial injury (200x).

Wire injury was conducted at Day 4. Neointima was not developed at Days 7 and 14. Neointima was formed at Day 18, and neointimal hyperplasia was obviously observed at Day 29. Endothelial regeneration was assessed by CD31 positive area. CD31 positive cells covered most part of the lumen at Day 14, and the coverage was completed at Day 18. Arrows indicate neointima and arrowheads show CD31 positive cells.

Fig. S4. Effects of liraglutide on body weight changes and fasting plasma glucose levels in db/db mice. Mice were treated with vehicle or liraglutide (17 or 107 nmol kg^-1^ d^-1^) for 28 days.

a, Body weight changes from baseline. b, Fasting plasma glucose levels. n = 3– 5. *, p < 0.05.
